# Recent Advances in Electrochemical Detection of Cell Energy Metabolism

**DOI:** 10.3390/bios14010046

**Published:** 2024-01-15

**Authors:** Kyeong-Mo Koo, Chang-Dae Kim, Tae-Hyung Kim

**Affiliations:** School of Integrative Engineering, Chung-Ang University, 84 Heukseuk-ro, Dongjak-gu, Seoul 06974, Republic of Korea; sse0913@cau.ac.kr (K.-M.K.); rlackdeo4@cau.ac.kr (C.-D.K.)

**Keywords:** electrochemical biosensor, glycolytic metabolites, mitochondrial metabolites, cell chip, wearable sensor

## Abstract

Cell energy metabolism is a complex and multifaceted process by which some of the most important nutrients, particularly glucose and other sugars, are transformed into energy. This complexity is a result of dynamic interactions between multiple components, including ions, metabolic intermediates, and products that arise from biochemical reactions, such as glycolysis and mitochondrial oxidative phosphorylation (OXPHOS), the two main metabolic pathways that provide adenosine triphosphate (ATP), the main source of chemical energy driving various physiological activities. Impaired cell energy metabolism and perturbations or dysfunctions in associated metabolites are frequently implicated in numerous diseases, such as diabetes, cancer, and neurodegenerative and cardiovascular disorders. As a result, altered metabolites hold value as potential disease biomarkers. Electrochemical biosensors are attractive devices for the early diagnosis of many diseases and disorders based on biomarkers due to their advantages of efficiency, simplicity, low cost, high sensitivity, and high selectivity in the detection of anomalies in cellular energy metabolism, including key metabolites involved in glycolysis and mitochondrial processes, such as glucose, lactate, nicotinamide adenine dinucleotide (NADH), reactive oxygen species (ROS), glutamate, and ATP, both in vivo and in vitro. This paper offers a detailed examination of electrochemical biosensors for the detection of glycolytic and mitochondrial metabolites, along with their many applications in cell chips and wearable sensors.

## 1. Introduction

Cells play dynamic roles in all physiological activities that sustain the human body [1,2]. The proper functioning of cells relies on the maintenance of their cellular homeostasis, pH, and ion concentrations [3,4,5]. Investigating fundamental biomolecules and biochemical activities, such as energy metabolism, can help reveal key biological events in various diseases and aid in the successful development of innovative diagnostic and therapeutic approaches [6,7,8]. To this end, there has been widespread integration of clinical monitoring and diagnostic techniques capable of providing high sensitivity, specificity, and rapid detection [9,10,11]. The integration of conventional biological ideas with digital devices has been facilitated by recent advancements in bioanalytical methods, resulting in the development of user-friendly portable systems, such as biosensors [12,13].

A biosensor is an analytical device for identifying and measuring analytes, including numerous cellular energy metabolites, such as glucose (Glc), lactate, nicotinamide adenine dinucleotide (NADH), reactive oxygen species (ROS), glutamate, and adenosine 5′-triphosphate (ATP) [14,15,16,17,18,19,20,21]. Biosensors measure changes in biological processes, and the transducer component of the biosensor converts the stimulus into a measurable electrical or light signal. For the detection of chemical compounds or cells, electrical and optical signals are combined with specific biological reactions, such as the isolation of enzymes, tissues, or whole cells [22,23,24,25]. Given the complex structure of the processes inside cells, conventional tools like the Clark oxygen electrode and glucometer are employed to monitor cell energy levels with exceptional precision [26]. However, despite their demonstrated efficacy, they still have drawbacks in terms of time consumption and intrusive methodology in certain situations. In order to overcome these challenges, numerous studies have proposed and devised electrochemically (EC) based biosensors capable of precisely detecting intact cells and their intricate interactions inside complicated biomolecular systems; these have been reviewed elsewhere [27,28,29], providing valuable insights in the fields of medical research and diagnostic medicine because of their ability to detect dynamic changes in cellular activity [30,31,32].

Recent advancements in EC cell-based biosensors have prompted numerous investigations and explorations. Several EC biosensor platforms, such as electrical cell–substrate impedance sensing, amperometric, and potentiometric sensors, have been developed and implemented for improved sensitivity and selectivity [33,34]. In the context of EC sensing-based biosensors, the biosensor detection procedure exhibits cost-effectiveness and enhanced speed compared to conventional techniques [35]. Hence, it exhibits favorable performance characteristics for point-of-care (POC) applications, namely in terms of label-free operation and straightforward downsizing. Numerous research investigations have shown an increase in the sensitivity and selectivity of biosensing platforms, suggesting the feasibility of reducing the limit of detection (LODs) of sensors for specific target analytes [36,37,38,39]. A notable development is an EC biosensing platform for detecting cell energy metabolism [40,41,42]. This platform has the potential to diagnose diabetes mellitus and Parkinson’s disease, among several other metabolic or mitochondrial disorders [43,44,45].

Cellular energy metabolism is a dynamic process involving ions, metabolic intermediates, and products generated by biochemical processes, notably glycolysis and mitochondrial metabolism [46,47,48]. Impaired cellular energy metabolism and metabolic perturbations or dysfunctions are frequently implicated in numerous pathological diseases, such as diabetes, cancer, and neurodegenerative and cardiovascular disorders. As a result, such impairments hold value as potential disease biomarkers, and their detection is imperative to early detection and treatment.

**Figure 1:**
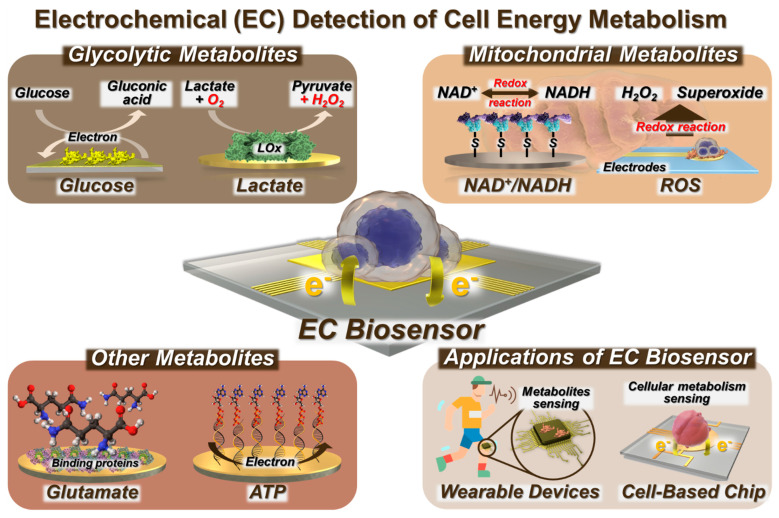
Schematic illustration of EC biosensors for cell metabolism detection and their applications.

Glycolysis is a fundamental metabolic path that takes place within the cytoplasm of cells [49,50]. It involves the enzymatic conversion of Glc into pyruvate and lactate. Mitochondria are the main sites for oxidative phosphorylation (OXPHOS) and ATP synthesis through various biomolecules, such as NADH and glutamate [51,52]. Consequently, there has been an increase in the application of EC biosensors to examine cellular energy metabolism and associated metabolites, including glycolytic metabolites, such as Glc and lactate and mitochondrial metabolites like NADH and ROS, as well as various metabolites involved in metabolic reactions. NAD^+^ is the oxidized form of NADH, and nicotinamide adenine dinucleotide phosphate (NADPH) is the reduced form of NADP^+^. As NAD^+^/NADH and NADP^+^/NADPH are redox couples, EC biosensing may be applied to the evaluation of metabolic processes, specifically glycolysis and OXPHOS, by analyzing their redox reactions (Figure 1).

EC sensors have found practical applications through the development of cell chips and wearable sensors. EC biosensing enables the effective implementation of early diagnosis and treatment strategies while minimizing any potential harm to living cells and the human body [53].

Here, we present a number of review papers that focus on the detection of cells via immobilization techniques and the sensing of metabolites utilizing biosensors. Nevertheless, this review encompasses not only biosensors designed for the detection of metabolites but also the identification of live cells via analysis of their cellular energy metabolism. This research specifically focuses on a range of biomolecules, such as metabolites, and expands its scope to include a whole cell and its application in wearable devices. Furthermore, this review could emphasize the progress achieved in the field of EC biosensors for the monitoring of cell energy metabolism. Additionally, the applications of these biosensors, such as their application in detecting live cells and their integration into cell chips and wearable sensors, are discussed (Figure 1). In addition, we provide a comprehensive analysis of a biosensor framework for diverse cell metabolites from a diagnostic perspective.

## 2. EC Biosensors for Glycolytic Metabolite Detection

Glc detection has an important role in healthcare and the food industry by ensuring safety and maintaining quality standards [54,55]. In addition, precise and continuous Glc monitoring is required for optimal management of diabetes and to reduce the occurrence of diabetes-associated complications. Therefore, Glc serves as a crucial biomarker for both type 1 and type 2 diabetes, necessitating the implementation of an efficient strategy for Glc monitoring [56].

Over several decades, researchers have made notable advancements in the development of several methods for Glc detection, including both biological and chemical detection methods [57,58]. In this regard, EC sensors have emerged as the preferred tool owing to their inherent advantages, such as their notable sensitivity and straightforward operational procedures. In the context of non-enzymatic biosensors, however, it has been shown that the surface of a nanostructured material-modified electrode is susceptible to pollution and passivation, eliminating the ability to selectively detect Glc. Hence, the pursuit of stable Glc biosensors continues to promote the exploration of novel approaches to mitigate the effects of temperature fluctuations. In this section, we discuss EC biosensors for the detection of glycolytic metabolites (Table 1).

### 2.1. EC Detection of Glc

Recently, electrochemical approaches such as amperometry and cyclic voltammetry (CV) have been investigated for Glc detection using various functional and metal electrodes [59,60,61,62,66,67] to enhance the sensitivity and stability of biosensor performance. Nevertheless, constant barriers remain in the pursuit of establishing a reliable detection system that can withstand temperature fluctuations. These challenges primarily stem from the susceptibility of the Glc-related enzyme to deactivation, the limited ability of detection techniques to mitigate interference, and the impact of external environmental factors.

**Figure 2:**
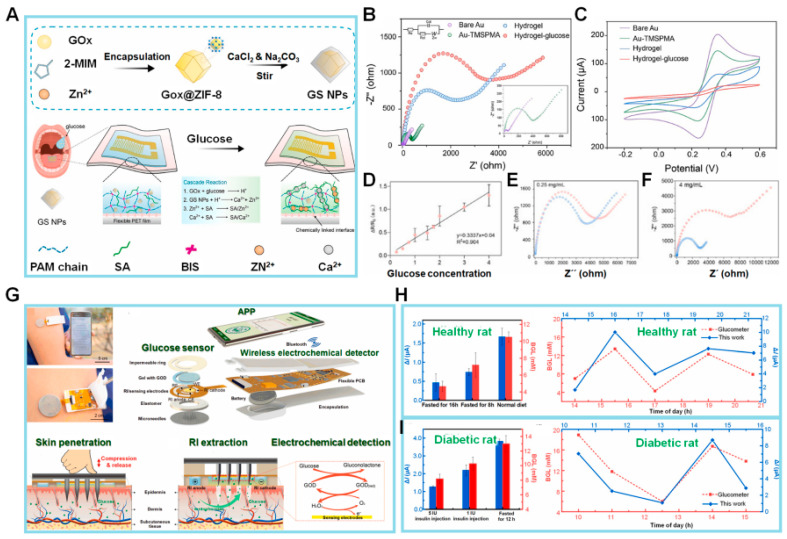
(**A**) Schematic illustration of the design of GOx@ZIF-8/CaCO_3_ (GS NPs) and the flexible hydrogel-based biosensor. (**B**,**C**) EIS and CV graphs of different modified electrodes in 5 mM [Fe(CN)_6_]^3−/4−^ containing 0.1 M KCl. Inset: Magnified view of EIS of bare Au electrode and the modified electrode, Au-TMSPMA. (**D**–**F**) Calibration curve of biosensor for glucose (Glc) vs. Glc concentration. EIS results of the biosensor response to Glc concentrations ranging from 0.25 to 4.0 mg/mL (Blue: EIS before Glc addition, Red: EIS after Glc addition). (**G**) The concept of smartphone-based glucose electrochemical detection platform (GEDP) integrated microneedle array (MA) with reverse iontophoresis (RI) technique for the minimally invasive detection of Glc in interstitial fluid (ISF). (**H**,**I**) The static current differences (Δ*I*) detected via the smartphone-based GEDP and the blood glucose levels (BGLs) measured by a commercial blood glucometer in healthy rats and diabetic rats. Dynamic chronoamperometric Δ*I* detected via same platform vs. BGLs measured in the tail vein blood using a commercial glucometer. (Reprinted with permission from [68]. Copyright 2022, Elsevier; reprinted with permission from [70]. Copyright 2022, Elsevier).

Wang et al. [68] presented a proof-of-concept hydrogel-based EC impedimetric biosensor for Glc detection within a temperature range of 20–60 °C, although the resistance to charge transfer (R_ct_) value decreased slightly with increasing temperature (Figure 2A). The biosensor constructed by those authors is composed of a hydrogel matrix, glucose oxidase (GOx) as a probe protein to detect Glc, a mineralizer to induce biomineralization, and a protective layer consisting of a metal–organic framework of zeolite imidazole framework-8 and biomineralized calcium carbonate (ZIF-8/CaCO_3_) to protect GOx from harsh environments (e.g., organic solvents and high temperature). The ZIF-8/CaCO_3_ layer is further immobilized in a three-dimensional (3D) hydrogel network composed of sodium alginate (SA) and polyacrylamide (PAAm). The combination of biomineralized GOx@ZIF-8/CaCO_3_ and 3D hydrogel networks has been shown to enhance the chemical stability of enzyme-based biosensors commonly used in various applications. The proposed biosensor exhibits higher selectivity toward Glc than other interfering molecules, such as fructose, maltose, sucrose, galactose, glutamate, quinine, and NaCl.

Several steps are involved in constructing an EC biosensor, but they typically include electrode preparation, electrode modification (often with nanostructures), and biological element immobilization. In the study described above by Wang et al. [68], electrochemical impedance spectroscopy (EIS) measurements and CV analysis were conducted to evaluate the stepwise construction process of their biosensor in 0.1 M KCl solution containing 5 mM [Fe(CN)_6_]^3−/4−^. Figure 2B displays Nyquist plots in which the impedance spectrum contains a semicircular component denoting the charge transfer mechanism alongside a linear segment that corresponds to the diffusion process. The R_ct_ is equivalent to the diameter of the semicircle, and its value is influenced by the type of chemicals used to modify the electrode. EIS measurements revealed rapid electron transfer kinetics of the unmodified gold (Au) electrode, as demonstrated by the relatively small semicircular and discernible linear segment. When the Au surface was chemically modified via the attachment of 3-(trimethoxysilyl)propyl methacrylate (TMSPMA), the resulting curve exhibited a wider semicircle compared to bare Au due to the weak conductivity of TMSPMA. There was a gradual rise in impedance after the immobilization of the bioreceptor in the hydrogel and a further increase after the successful capture of Glc and the synthesis of suboptimal conductive SA gels, hence demonstrating the proficient production of the desired biosensor.

The results from the CV analysis seen in Figure 2C supported the ESI findings. The modified electrode exhibited a diminished current (*I*) signal compared to the unmodified electrode because of its reduced conductivity, and the *I* signal decreased further after modification of the electrode with hydrogel because SA gel that formed in situ further hindered the diffusion of ferrocyanide ions from the bulk solution to the electrode surface. Furthermore, the peak potential separations (ΔE_p_) were comparable to those seen with the unmodified electrode. Collectively, the CV and EIS data suggested the successful fabrication of the hydrogel-based biosensor and the efficient detection of Glc.

Prior to determining the analytical range of the biosensor, the authors examined the impact of incubation duration in Glc presence on achieving optimal biosensor performance. Immobilization of the biosensor was achieved after exposure to Glc for 120 min. The R_ct_ values of the developed impedimetric biosensor were determined at Glc concentrations ranging from 0.25 to 4.0 mg/mL. Figure 2D exhibits the correlation between the change in resistance (ΔR/R_0_) and the concentration of Glc. These measurements are depicted in Figure 2E,F: here, ΔR represents the difference between the resistance of the system (R_ct_) before and after the introduction of Glc, which is calculated as ΔR = R_cti_ − R_ct0_. The fitting curve, which could be described by the equation *y* = 0.337*x* + 0.04, was constructed using the percentage change in R_ct_ values derived from the impedance spectra. The Glc molecules that effectively permeated the hydrogel were then catalyzed with GOx to trigger a cascade reaction, resulting in an increase in impedance values. This artificial hydrogel biosensor represents an appropriate approach for Glc detection.

In the context of Glc monitoring, the challenge of developing a non-invasive biosensor for monitoring Glc in interstitial fluid (ISF) persists due to limitations in the extraction of glucose from ISF by standard reverse iontophoresis (RI), which is characterized by low extraction flux and inconsistency [69]. Cheng et al. developed a touch-actuated biosensor for measuring Glc levels in ISF directly based on a sensing strategy consisting of skin penetration, RI extraction, and EC detection, as seen in Figure 2G [70]. The sensor has three main components: a solid microneedle array (MA) for painless skin penetration, an RI unit for ISF extraction through the MA-coated microchannels, and a sensing unit for measuring Glc. The skin penetration–RI extraction sampling technique improved Glc extraction by about 1.6 times compared to RI extraction alone, both in vitro and in vivo, showing that Glc extracted from ISF by RI enhanced the capacity of the biosensor to detect Glc levels. These findings align with earlier research conducted by Chen et al. [71] and Lipani et al. [72].

In the same study by Cheng et al. described above, the authors devised a glucose electrochemical detection platform (GEDP) for smartphones. This platform consists of a touch-actuated Glc sensor, a wireless EC detector, and a customized Android-based smartphone application designed specifically for Glc sensing in vivo. The performance of the GEDP was compared to a commercial blood glucometer as the control by in vivo experiments using three distinct groups of healthy rats: rats which fasted for 16 h but with ad libitum access to liquid; rats which fasted for 8 h; and rats on a regular diet without any fasting. Additionally, the chronoamperometric response curves of three distinct groups of rats with diabetes were measured: rats injected with 5 IU insulin, rats injected with 1 IU insulin, and rats which fasted for 12 h. Figure 2H,I demonstrate the disparities in the measured current (Δ*I*) between the rats’ blood Glc levels recorded via the commercial blood glucometer and the smartphone-based GEDP. The two results from the tests exhibited a strong correlation, suggesting that the smartphone-based GEDP is capable of accurately detecting Glc levels in living organisms through the process of skin penetration, RI extraction, and EC detection. Therefore, this approach presents the potential to dynamically monitor a diverse array of biomarkers in the ISF.

Future research may prioritize enhancing the precision and responsiveness of glucose sensors to enable more effective glucose monitoring, especially in critical medical contexts like diabetes management. Additionally, there may be a focus on developing new materials and coatings that improve the compatibility of sensors and extend their lifespan. Advancements in microfabrication and nanotechnology have the potential to enable the development of increasingly compact and discreet wearable glucose sensors. By allowing glucose sensors to simultaneously detect additional analytes, such as lactate, it would be possible to obtain comprehensive real-time health information. Exploring non-invasive or minimally intrusive methods, such as using saliva or tears to measure glucose levels, could offer more convenient options for monitoring glucose. Potential advancements in sensor technology could incorporate wireless communication technology to transmit data to smartphones or cloud platforms. This would enable remote monitoring and real-time data exchange with healthcare professionals. The development of implanted sensor technology may facilitate long-term glucose monitoring within the body, providing assistance to patients who require continuous monitoring.

### 2.2. EC Detection of Lactate

Lactate has typically been regarded as a byproduct of cellular metabolism or a source of energy [73]. However, recent research has shed light on the role of lactate as an important signaling molecule in numerous physiological and pathological conditions [74,75]. Lactate plays a crucial function in facilitating tumor cell metastasis, angiogenesis, and treatment resistance, besides providing an energy source for tumor cells [76]. Lactate has a crucial role in regulating several neuronal activities, such as excitability, plasticity, and consolidation of memories, in addition to its metabolic activities in brain tissue [77].

Various analytical techniques, including EC sensing and colorimetric analysis, have been employed to quantify lactate concentrations. Enzymes, such as lactate oxidase (LOx), are commonly used as biorecognition elements in EC biosensors. Nevertheless, for redox enzymes, such as flavin adenine dinucleotide (FAD)-based enzymes with their active sites deeply buried in the protein structure, the transfer of electrons from the redox center to the electrode is impeded. In recent years, EC techniques, including amperometry and cyclic voltammetry (CV), have been commonly employed for lactate detection. Commonly, the electrode surface is chemically modified using various polymers for lactate immobilization and sensitive detection. Additionally, the limit of detection (LOD) and sensitivity varies depending on the electrodes utilized [63,64,65,66].

In the field of EC biosensors, an increased specific surface area might yield a greater number of active sites, hence resulting in the amplification of electrical signals. In this context, a two-dimensional polyaniline (2D PANI) structure has the potential to improve the sensitivity of biosensors appreciably. The fabrication of 2D PANI films is typically conducted using a graphene oxide (GO) template. The oxygen groups present in GO are chemically bonded with the amino groups of aniline, resulting in the growth of aniline molecules along the GO surface through π–π stacking interactions. This process leads to the formation of a 2D PANI structure. While it is possible to synthesize 2D PANI, the acquisition of an extensive area of PANI film using this technique is challenging. Additionally, there are some drawbacks associated with its use, including high cost and a complicated synthesis procedure. Hence, there is a requirement for a straightforward technique to fabricate a PANI film devoid of templates.

**Figure 3:**
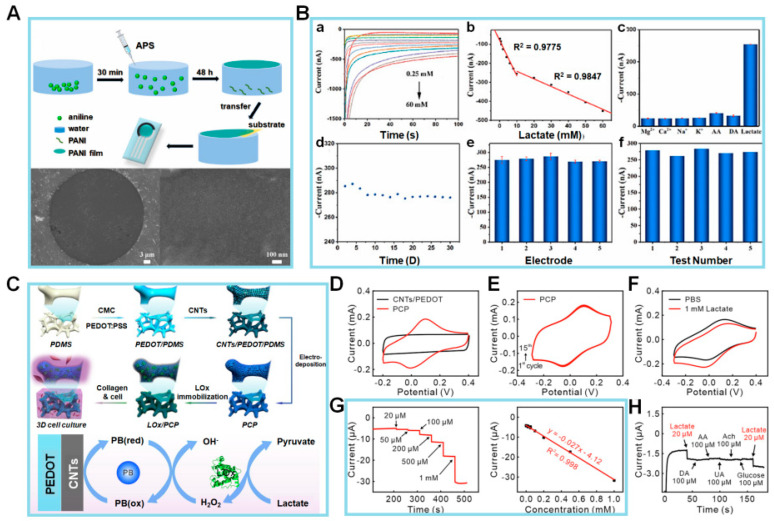
(**A**) Schematic illustration of PANI film (above) and SEM images (below). (**B**) Amperograms (**a**) and calibration curve (**b**) of PANI-3/SPCE to 0.25 to 60 mM lactate in phosphate buffer; peak current (*I*) of PANI-3/SPCE to 10 mM interference substance and lactate (**c**); peak *I* of PANI-3/SPCE during 30 days storage (**d**); applied potential: −0.15 V; the reproducibility tests with five electrodes (**e**) and five consecutive (**f**) measurements of PANI-3/SPCE to 20 mM lactate. (**C**) Schematic diagram of the fabrication processes of the 3D LOx/PCP electrochemical sensor and collagen hydrogel integrated platform (above) and the mechanism of lactate detection on this platform (below). (**D**) Cyclic voltammetry (CV) for PBS recorded at PCP (red) and CNTs/PEDOT (black) electrodes. (**E**) CV results obtained at PCP electrode in PBS (pH 7.0) for 15 cycles. (**F**) CV results for PBS with (red) and without (black) 1 mM lactate at LOx/PCP electrode (scan rate, 10 mV/s). (**G**) Amperometric responses of LOx/PCP electrode to a series of increasing lactate concentrations in PBS solution at a potential at −0.05 V (vs. Ag/AgCl). Corresponding calibration curve of the electrode in lefthand panel. (**H**) Amperometric responses to 20 µM lactate and potential interfering agents at 100 µM concentration on proposed platform. (Reprinted with permission from [78]. Copyright 2022, Elsevier; reprinted with permission from [79]. Copyright 2022, Elsevier).

Zhu et al. [78] synthesized a PANI film with a well-organized structure using an air–water interfacial synthesis approach, as seen in Figure 3A. The PANI film, used for detecting pH and lactate levels in sweat, is screen-printed onto a flexible substrate using a screen-printed electrode (SPE). The aniline monomer diffuses into the ammonium peroxydisulfate solution, resulting in the formation of a PANI film upon contact with water. In contrast, free aniline oligomers undergo precipitation and do not actively contribute to the film production process. The PANI film was placed on a copper mesh, and its morphology was examined via scanning electron microscopy (SEM). The SEM images showed that the surface of the PANI film exhibited a flat morphology, allowing it to be suspended within a circular aperture of 40 µm in diameter. Hence, it can be concluded that the suggested sensor indicates that the PANI film possesses a *p2gg* plane group with a rectangular lattice characterized by long chains. The unit cell characteristics of the film were found as follows:

*a* = 6.9 Å, *b* = 7.6 Å, *α* = 97°, *β* = *γ* = 90°

Following the successful manufacturing process of the sensor, a comprehensive investigation was conducted to analyze its EC characteristics based on lactate detection. This was important because changes in the lactate concentration of sweat can influence the pH value. The chronoamperometric-response *I* of the PANI–screen-printed carbon electrode (SPCE) sensor to lactate concentrations ranging from 0.25 to 60 mM at a potential of −0.15 V is depicted in Figure 3B(a). It displayed a linear response that can be divided into two distinct ranges: 0.25–10 and 10–60 mM. The corresponding sensitivities for these ranges are 18.62 and 4.25 nA/mM, respectively (Figure 3B(b)). Furthermore, the sensor demonstrates an LOD of 0.083 mM. The sensor’s selectivity for lactate is 10 mM in the presence of interfering chemicals, including Mg^2+^, Ca^2+^, Na^+^, K^+^, ascorbic acid (AA), and dopamine (DA), each at a concentration of 10 mM. The findings indicate that the sensor remains unaffected even in the presence of other chemicals (Figure 3B(c)). To examine its durability, the storage stability of the fabricated sensor was assessed every 2 days for a total of 30 days, as seen in Figure 3B(d). In comparison to the starting *I*, the *I* exhibits minimal fluctuations, with a low relative standard deviation (RSD) value of 1.29%, confirming its stability. When testing the analytical reproducibility of the PANI-3/SPCE sensor, a total of five sensors were employed for the detection of 20 mM lactate, which resulted in an RSD value of approximately 3.33%. Subsequently, five successive measurements of 20 mM lactate using the identical electrode were performed, and the relative RSD was determined to be 3.01% (Figure 3B(e,f)).

Collectively, these experimental findings demonstrate that the PANI-3/SPCE sensor exhibits favorable reproducibility and repeatability in detecting lactate. Whereas the lactate sensors described in the literature often immobilize LOx onto sensitive substrates, the manufactured PANI film described above does not include LOx. Despite the compromised sensitivity of the suggested sensor, there is a cost advantage for the synthesis. As a result, the PANI sensor was also employed for the detection of lactic acid. It showed excellent linearity, with a sensitivity of 18.62 and 4.25 nA/mM for lactic acid concentrations ranging from 0.25–10 and 10–60 mM, respectively. The PANI sensor effectively identified alterations in pH and lactic acid levels in sweat. Subsequent investigations will be directed toward enhancing the sensor’s overall efficiency and seamlessly incorporating it into a compact sensing system, hence facilitating its use in the realm of wearable sweat analysis.

A lactate-detecting EC sensor was designed by Yao et al. [79] as a 3D enzymatic biosensor and was successfully integrated with collagen hydrogel to enable the measurement of lactate levels in cultured cells, as seen in Figure 3C. In that work, the EC sensor was fabricated via electrodeposition of Prussian blue nanoparticles (PBNPs) on carbon nanotubes (CNTs) absorbed on the surface of a 3D conductive scaffold. The CNTs increased the specific surface area, and the purpose of the PBNPs was to enhance the electrocatalytic efficiency for the electrooxidation of hydrogen peroxide (H_2_O_2_). LOx was immobilized onto the electrode surface using a gentle crosslinking technique. In addition, the interspace of the 3D lactate EC sensor was filled with collagen hydrogel containing C6 glioma cells. This 3D lactate sensor for monitoring lactate release from glioma cells inside a collagen matrix enabled real-time in situ monitoring of biochemical responses in 3D cell culture systems, hence offering an effective platform. Subsequently, an assessment was conducted on the EC performance of the LOx/pro-collagen C proteinase (PCP) electrode. LOx oxidized lactate to yield pyruvate and H_2_O_2_. The resulting oxidation product, H_2_O_2_, was subsequently electrochemically reduced by the electrode through a Prussian blue-mediated process. Because of the susceptibility of the polycrystalline structure of PBNPs to disintegration in neutral or alkaline pH solutions, the stability of the electrode was initially assessed by conducting CV scans for a total of 15 cycles in PBS (pH 7.0). In comparison to the CV curve observed at the CNTs/poly(3,4-ethylenedioxythiophene) (PEDOT) electrode, the characteristic redox peaks of Prussian blue were observed throughout the potential range of −0.1 to 0.2 V (Figure 3D). This observation suggests the effective electrodeposition of PBNPs onto the PCP electrode. There was also a high level of consistency of the redox peaks over the 15 cycles, as depicted in Figure 3E. Furthermore, the CV curves at the Prussian blue/PEDOT electrode displayed a progressive attenuation and eventual disappearance. This observation serves as evidence for the crucial role performed by CNTs in stabilizing the structure of Prussian blue and enhancing its electrocatalytic performance.

Next, tests were conducted to assess the EC performance of the LOx/PCP electrode in the presence of lactate. The CV results depicted in Figure 3F show that the biosensor has a typical catalytic oxidation response, with a marked decrease in peak current (*I*_p_) at the LOx/PCP electrode in 1 mM lactate solution (red curve) in comparison to the curve obtained in PBS (black curve). In contrast, there was no discernible disparity observed in the CV curves obtained from the PCP electrode, regardless of the presence or absence of 1 mM lactate. The favorable electrocatalytic characteristics of the LOx/PCP electrode toward lactate can be attributed to the presence of LOx.

Subsequently, the authors conducted a more in-depth analysis of the electrocatalytic efficiency of the LOx/PCP electrode via the amperometric technique with a range of lactate concentrations (Figure 3G, lefthand panel). The experimental findings indicate that the LOx/PCP electrode has a rapid response for detecting lactate, with a positive correlation between the *I* and lactate concentration. The calibration curve was linear over the concentration range of 0.02–1 mM, as seen in Figure 3G (righthand panel). Additionally, the LOD for lactate was determined to be 0.75 μM, and the LOx/PCP electrode exhibited a high degree of selectivity toward lactate when compared to commonly encountered electroactive interfering species, like DA, AA, and uric acid (UA), as well as biologically relevant species, like acetylcholine (ACh) and Glc (Figure 3H). The observed advantages stemmed from the synergistic combination of LOx’s high selectivity and Prussian blue’s exceptional electrocatalytic capabilities. As the authors demonstrated, this combination enabled the accurate identification of lactate at a low potential of –0.05 V compared to Ag/AgCl. The stability of the lactate sensor was verified using the recurrent detection of 0.1 mM lactate solution over 12 days. Overall, the exceptional EC characteristics of the LOx/PCP electrode for lactate detection suggest its considerable potential for the continuous monitoring of lactate release from cells. In the context of the importance of aerobic glycolysis, the suggested 3D EC sensor is anticipated to function as a robust and adaptable instrument for investigating metabolic processes associated with lactate in cancer as well as neurons and other brain cells inside 3D cell culture systems.

In terms of utilization for the 3D system and the end of cellular activities, lactate detection was more widely utilized for diverse metabolic sensing in comparison to Glc detection. For our perspectives, future efforts will primarily concentrate on enhancing the sensor’s performance and integrating it into a compact sensing system that is suited for analyzing sweat in wearable devices.

## 3. EC Detection of the Mitochondrial Metabolites

Mitochondria, as cellular organelles responsible for the process of cellular respiration, participate in the consumption of oxygen through the respiratory chain [80]. In this process, redox energy is transformed into ATP, the primary provider of cellular energy. NADH, ROS, and glutamate are essential intracellular redox-active chemicals involved in this biological process [81,82]. These molecules also affect several pathological processes, including inflammation, neurological disorders, and cancer, because of their important role in maintaining cellular redox homeostasis [83].

In addition, there are several obstacles to real-time and accurate identification of mitochondrial energy metabolites (such as NADH, ROS, glutamate, and ATP), both within living organisms and in laboratory settings. These challenges encompass factors such as low concentration levels, short activity periods, facile conversion, and interference from external metabolites. Hence, it is imperative to diligently monitor the dynamics of mitochondrial metabolism by employing a diverse array of EC biosensors that provide accuracy, sensitivity, and selectivity. This section will provide a comprehensive analysis of the EC biosensors employed for the identification of mitochondrial metabolism, as stated in Table 2.

### 3.1. EC Detection of NAD^+^/NADH

NADH and NAD^+^ are cofactors, carry electrical charges, and perform essential functions in fundamental metabolic pathways, such as OXPHOS, the tricarboxylic acid cycle, and glycolysis [92]. Additionally, they contribute to the preservation of cellular oxidation, cell signaling, and DNA repair [93]. One example is the role of NADH in promoting the synthesis and activation of neurotransmitters within cells, thereby enhancing both muscle coordination and cognitive focus. ATP depletion and NAD^+^/NADH ratio imbalance are implicated in Parkinson’s disease. In the context of breast cancer cells, the NADH concentration has been estimated to be 168 ± 49 µM, which represents a 1.8-fold increase when compared to the NADH concentration in normal breast cells. Therefore, the sensitive, selective, and real-time monitoring of NADH is essential for screening different disease states and tracking their therapeutic progress.

**Figure 4:**
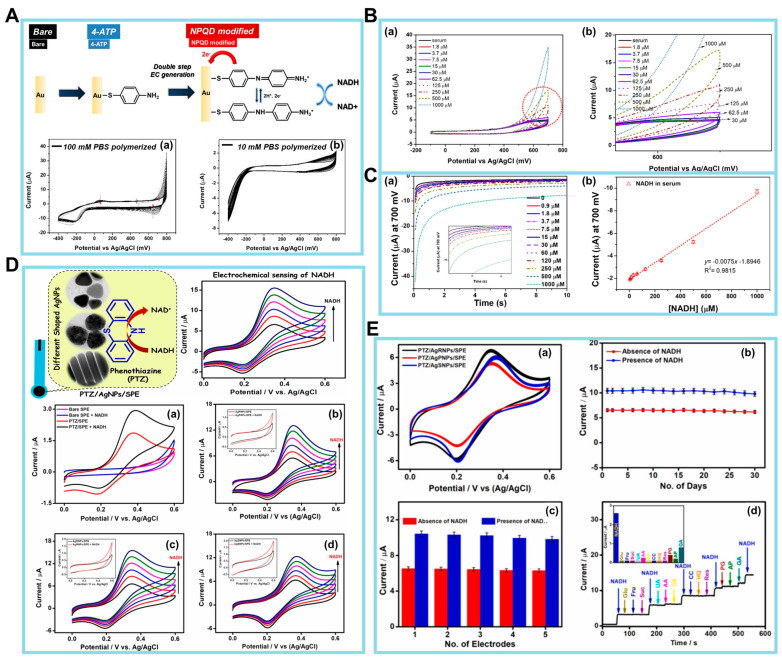
(**A**) Schematic diagram of NPQD modification (upper panel). Cyclic voltammetry (CV) data obtained during the electrochemical functionalization of the 4-aminothiophenol Au electrode in (**a**) 100 mM PBS (pH 7.4) and (**b**) 10 mM PBS (pH 7.4). The scan rate is 100 mV/s. (**B**) Electrochemical results for NADH in mouse serum. (**a**) Voltammogram results (potential range: from −100 to 700 mV). (**b**) CV from the circled part of graph (**a**). (**C**) (**a**) NADH quantification measured via CA. (**b**) Calibration plot of NADH in mouse serum (0–1000 µM). The current at 10 s in the CA graph was used for NADH quantification. (**D**) (**a**) CVs of bare SPE and PTZ/SPE in the absence and presence of 100 μM NADH. (**b**) PTZ/AgPNPs/SPE (**c**) PTZ/AgRNPs/SPE, and (**d**) PTZ/AgSNPs/SPE with sequential additions of each 20 μM NADH (20–100 μM). Insets of (**b**–**d**) are CVs of AgPNPs/SPE, AgRNPs/SPE, and AgSNPs/SPE in the absence and presence of 100 μM NADH. Electrolyte: 0.1 M phosphate buffer solution (pH 7.0) at a scan rate of 20 mV/s. (**E**) (**a**) CVs of PTZ/AgPNPs/SPE, PTZ/AgRNPs/SPE, and PTZ/AgSNPs/SPE for 50 continuous scans at the scan rate of 20 mV/s. (**b**,**c**) Long-term stability and reproducibility study of PTZ/AgRNPs/SPE in the absence and presence of 40 µM NADH. (**d**) Amperometric response of PTZ/AgRNPs/SPE to 15 µM NADH in the presence of 75 µM of various interfering species in 0.1 M phosphate buffer solution at an applied potential of +0.4 V. (Reprinted with permission from [94]. Copyright 2022, Springer Nature; reprinted with permission from [95]. Copyright 2021, Elsevier).

The preservation of homeostasis in live cells is dependent upon the maintenance of mitochondrial function. Mitochondria are integral in facilitating essential metabolic activities that sustain life, including energy conversion and calcium signaling within biosynthetic pathways. In recent years, electrochemical biosensors for NADH have emerged as a viable alternative for conventional analytical methods, such as absorbance or fluorescence-based optical assays. The amperometry method, a type of electrochemical (EC) approach, was utilized for the detection of NADH. In this case, a functional material was employed to enhance the sensitivity of the detection process [84]. Next, the NADH/NAD^+^ redox couple was employed in the sensor designed by Lee et al. [94], who introduced a two-step EC functionalization technique to address the challenges encountered in the measurement of body fluids. For NADH detection, a disposable electrocatalytic sensor was constructed based on an SPE coated with a redox-active monolayer of 4′-mercapto-N-phenylquinone diamine (NPQD) formed via self-assembly of 4-aminothiophenol, as depicted in Figure 4A (upper panel). It is noteworthy that the voltage, which is roughly −0.4 V, has minimal variation. This indicates that the outcomes obtained are consistent, and the alterations found in 100 mM PBS were not seen in 10 mM PBS (Figure 4A, lower panels). The electrocatalytic NADH oxidation of this platform was thoroughly examined, revealing that the electrode had an LOD of 3.5 µM and a sensitivity of 0.0076 ± 0.0006 µM/µA when tested in mouse serum. The electrocatalytic activity of the NPQD-Au electrode for NADH oxidation in the cell culture medium is demonstrated in Figure 4B, serving as evidence for the sensitivity and selectivity of the NADH sensor. The electrocatalytic sensor enables the direct investigation of mitochondrial malfunction by monitoring the NADH concentration within the cell. The CV results obtained from the mouse serum exhibit a resemblance to the signal observed in the culture medium, thereby providing evidence that the NADH sensor is functional in both environments (a,b). In addition, a driving force is a prerequisite for facilitating the oxidation of NADH to NAD^+^. It is observed that the I remain constant as the concentration of NADH increases within the range of −100 to 400 mV. To achieve a short and consistent potential analysis time per sample, chronoamperometry rather than CV was used to construct a calibration plot for NADH. According to the data presented in Figure 4C, the I remains constant at 10 s. Additionally, the I at this specific time point was determined by varying the quantities of NADH (a). Subsequent measurement of the NADH quantity in mouse serum exhibits a linear range spanning roughly 16–1000 µM, with an LOD of 3.5 µM (b). Notably, the sensitivity of the sensor in question was enhanced using a double-step electro-functionalization technique, as opposed to the single-step functionalization method. This improvement is seen from a lower LOD of 3.5 µM compared to the LOD of 10 µM. As a proof of concept, the sensor was applied to monitor the ex vivo release of NADH in blood samples collected from mice with polyhexamethylene guanidine phosphate-induced lung inflammation and fibrosis. The data obtained from the ex vivo tests provided evidence supporting the effectiveness of the NADH sensor. Nevertheless, it is imperative for future efforts to concentrate on establishing a comprehensive understanding of the NADH mechanism and its effect on the development of lung disease induced with polyhexamethylene guanidine phosphate.

Manusha et al. synthesized silver nanoparticles (AgNPs) with varying shapes, such as nanorods (AgRNPs), nanoprisms (AgPNPs), and nanospheres (AgSNPs) [95]. These produced AgNPs were then used in the development of an NADH sensor, which demonstrated both simplicity and high sensitivity. The EC sensor under consideration was constructed by immobilizing individual phenothiazine (PTZ) redox mediators on AgNPs-modified SPE nanohybrids. In the electrode fabrication process, PTZ played a key role as a redox mediator, and the AgNPs contributed to the enhancement of the overall performance of the sensor. Additionally, the shape-dependent catalytic performance of PTZ/AgNPs/SPE-modified electrodes was determined using CV and amperometry techniques. This study also evaluated analytical factors, including practical applicability, stability, and selectivity. An initial assessment of the electrodes’ electrocatalytic activity toward NADH oxidation was conducted using CV at a potential range from 0 to +0.6 V, with a scan rate of 20 mV/s. Data were recorded using bare SPE and PTZ/SPE both in the absence and presence of 100 µM NADH in 0.1 M phosphate buffer. These data are illustrated in Figure 4D(a). The SPE method did not demonstrate any notable reaction, even when NADH was present. Nevertheless, a decrease in the catalytic *I* response was seen during the electrocatalytic oxidation of NADH at the PTZ/SPE, commencing at around +0.25 V.

Subsequently, the catalytic performances of the PTZ/AgNPs/SPE, PTZ/AgRNPs/SPE, and PTZ/AgSNPs/SPE nanohybrids based on the oxidation of NADH were determined in parallel to control experiments without the PTZ redox mediator. The outcomes of the examination are depicted in Figure 4D(b–d). Based on the results of these experiments, it can be inferred that PTZ/AgNPs/SPE-manufactured electrodes have the potential to serve as a viable option for the detection of NADH.

Additional tests were carried out to determine the stability and repeatability of the constructed PTZ/AgNPs/SPE nanohybrid sensor via the analysis of voltammograms obtained from 50 consecutive scans, with a scan rate of 20 mV/s, as seen in Figure 4E(a). It is seen from these results that there is a lack of significant alteration in redox peak potentials, *I*_p_, and peak behavior, thus demonstrating the exceptional stability of the nanohybrid-modified sensors. To ascertain the enduring stability, the PTZ/AgRNPs/SPE system was monitored periodically over 30 days, both in the absence and presence of NADH. The outcomes of this investigation are shown in Figure 4E(b). The experimental results indicate that 92.07% of the *I* response was maintained in the presence of 40 µM NADH and 94.71% in the absence of NADH, even after 30 days. Moreover, the repeatability of the electrode-to-electrode measurements was assessed in the presence and absence of NADH. This was done using five distinct electrode conditions that were created under similar experimental settings. The outcomes of these measurements are presented in Figure 4E(c). The oxidation *I*_p_’s have an RSD of 1.56% and 2.48% in the absence and presence of 40 µM NADH, respectively. This indicates that the nanohybrid electrode that was constructed demonstrates remarkable repeatability for the detection of NADH.

Finally, a series of interference tests specifically focusing on the selectivity of the PTZ/AgRNPs/SPE modified electrode were carried out in the presence of 15 μM NADH and various electroactive interferents, including UA, AA, DA, Glc, fructose, sucrose, catechol, resorcinol, hydroquinone, pelargonin (PG), acetaminophen, and gallic acid (GA), each at 75 μM. The amperometric response represented in Figure 4E(d) demonstrates a notable *I* response with the addition of NADH. However, no significant alteration in the *I* response was seen for most of the interferents, except for AA, DA, PG, and GA. The interference caused by AA and DA had insignificant effects on the NADH response. In contrast, PG and GA demonstrated *I* responses of 14% and 30%, respectively. Therefore, the PTZ/AgRNPs/SPE-based sensing approach has demonstrated its effectiveness in detecting NADH in pharmaceutical samples. However, when detecting NADH in food samples, sample pretreatment may be required to remove interference from polyphenolic chemicals, such as GA and PG. There is future potential for the development of chemical biosensors that possess enhanced analytical properties by modifying the nanoparticle shape.

The overall results suggest that there are currently multiple valid methods to achieve efficient electrocatalysis of NADH on durable electrodes. The selectivity of the measurement is still a significant concern. However, when examining the use of dehydrogenase-based biosensors on actual samples, the challenges posed by interfering compounds have been and can be addressed through various strategies. These strategies include the use of protective membranes, standard additions, or blank subtraction, even for systems with low complexity (such as when a mediator is adsorbed onto the electrode surface). It is imperative for future research to focus on creating NADH sensing systems that preserve the characteristics of nanomaterials and nanostructured systems while also exhibiting a high level of biocompatibility. This will enhance the longevity of immobilized dehydrogenase enzymes.

### 3.2. EC Detection of Mitochondrial ROS

ROS, such as the superoxide anion (O_2_^•−^), hydroxyl radical (^•^OH), and H_2_O_2_, are key mediators in a range of physiological and pathological processes in living organisms [96]. Mitochondrial ROS mostly originate from the electron transport chain (ETC) inside mitochondrial complexes, namely complex I (ubiquinone oxidoreductase), complex II (succinate dehydrogenase), complex III (cytochrome c reductase), and complex IV (cytochrome c reductase) [97]. In live cells, the generation of ROS is typically counterbalanced by their removal under normal physiological circumstances. Nevertheless, this delicate equilibrium can occasionally be disrupted by stressors, such as an increase in metabolic rate, hypoxia, or damage to cellular membranes. In this context, it is imperative to observe ROS levels in viable cellular systems so that early detection of diseases can be achieved. However, the direct detection of O_2_^•−^ and ^•^OH within mitochondria is challenging due to the transient lives of these chemical species. Instead, the presence of ROS in mitochondria may be accurately measured by directly measuring the concentration of H_2_O_2_. Thus, researchers have successfully fabricated EC biosensors for the purpose of detecting mitochondrial ROS. Multifunctional frameworks, carbon-based materials, and transition metal-based materials are examples of some of the versatile biomaterials used for developing biosensors such as sensitivity and their stability [86,98,99].

**Figure 5:**
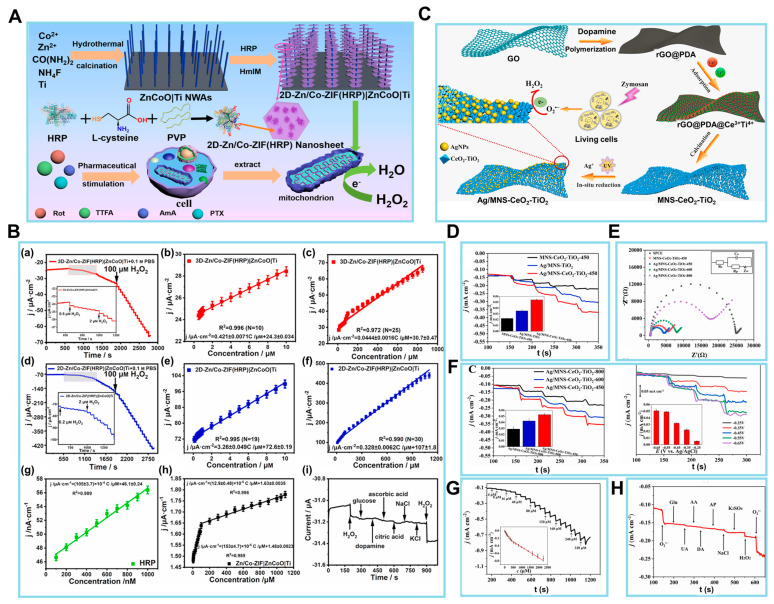
(**A**) Scheme of a 2D-Zn/Co-ZIF(HRP)|ZnCoO|Ti nanoarray biosensor for detecting H_2_O_2_ leakage from mitochondria after treatment with various inhibitors against the mitochondrial complexes. (**B**) Chronoamperometric results of increasing H_2_O_2_ concentration at (**a**) a 3D-Zn/Co-ZIF(HRP)|ZnCoO|Ti electrode. The corresponding calibration plots based on electrode surface area-normalized |steady-state current| in (**a**) in the low (**b**) and high (**c**) H_2_O_2_ concentration ranges. (**d**) A 2D-Zn/Co-ZIF(HRP)|ZnCoO|Ti. The corresponding calibration plots normalized |reduction peak current| in (**d**) in the low (**e**) and high (**f**) H_2_O_2_ concentration ranges. (**g**) Calibration plots based on |steady-state current| of HRP-modified GCE vs. H_2_O_2_ concentration. (**h**) Calibration plots of Zn/Co-ZIF|ZnCoO|Ti vs. H_2_O_2_ concentration. (**i**) Chronoamperometric responses of 2D-Zn/Co-ZIF(HRP)|ZnCoO|Ti obtained at −0.3 V via successive addition of H_2_O_2_ and various interfering agents. (**C**) Scheme of the synthesis of Ag/MNS-CeO_2_-TiO_2_ materials and the detection of cell-released superoxide anions (O_2_^•−^). (**D**) The *i–t* curve and bar graph (inset) of current (*I*) responses of MNS-CeO_2_-TiO_2_-450/SPCE, Ag/MNS-TiO_2_/SPCE, and Ag/MNS-CeO_2_-TiO_2_-450/SPCE at −0.55 V in 0.1 M Ar-saturated PBS toward the addition of 0.08 mM O_2_^•−^. (**E**) EIS curves in 0.1 M KCl solution containing 5 mM Fe(CN)_6_^3−/4−^. (**F**) The *i–t* curve and bar graph (inset) of amperometric responses toward 0.08 mM O_2_^•−^, and the *i–t* curve and bar graph of amperometric responses toward 0.08 mM O_2_^•−^ at each step in 0.1 M PBS (pH 7.4). (**G**,**H**) The amperometric responses and fitted linearity curve (inset) of Ag/MNS-CeO_2_-TiO_2_-450/SPCE toward different addition amounts of O_2_^•−^ in 0.1 M PBS (pH 7.4) at applied potential of −0.55 V. (**H**) *I* responses toward 0.08 mM O_2_^•−^, 4 mM Glu, 0.4 mM UA, 0.08 mM AA, 0.08 mM DA, 0.08 mM AP, 0.14 M NaCl, 4 mM K_2_SO_4_, 0.08 mM H_2_O_2_, and O_2_^•−^ added into 0.1 M PBS. (Reprinted with permission from [100]. Copyright 2023, Elsevier; reprinted with permission from [101]. Copyright 2021, Elsevier).

Jin et al. [100] developed a nanocomposite designated as 2D-Zn/Co-ZIF(HRP)|ZnCoO, which consisted of horseradish peroxidase (HRP)-encapsulated 2D Zn-Co ZIF nanosheets strung on a ZnCoO nanowire array on a Ti substrate (Figure 5A). This EC biosensor had the potential to detect H_2_O_2_ produced by O_2_^•−^ in human hepatocellular carcinoma (HepG2) cells. HepG2 is associated with a pathological condition characterized by oxidative stress resulting from an excessive accumulation of ROS. To minimize any disruptions caused by the cellular matrix, mitochondria were isolated from live HepG2 cells. Subsequently, four inhibitors of mitochondrial complexes, namely rotenone, 2-thenoyltrifluoroaceteone, antimycin A, and paclitaxel, were employed to specifically hinder the four primary sites of electron transfer in the mitochondrial ETC, and the O_2_^•−^ released from the compromised mitochondrial complexes was rapidly converted into H_2_O_2_ facilitated by the dismutase enzyme. These results demonstrate that the suggested sensor could indirectly monitor mitochondrial electron leakage. Furthermore, to investigate the charge/mass transfer properties of H_2_O_2_ in the nanocomposite, the 3D-Zn/Co-ZIF(HRP)|ZnCoO|Ti structure was converted to its 2D counterpart. Subsequently, the diffusion coefficient, charge transfer coefficient, and apparent charge transfer rate constant of H_2_O_2_ were estimated for both sensors.

The characterization of the EC biosensor involved the chronoamperometric assessment of 3D-Zn/Co-ZIF(HRP)|ZnCoO|Ti at a potential of −0.3 V. The results obtained, as depicted in Figure 5B, are presented in (a) and demonstrated two linear relationships, namely (b) and (c). In the range of 0.5 to 10 µM, the relationship (b) can be described as j/µA cm^−2^ = 0.421 ± 0.0071 C/μM + 24.3 ± 0.034 (R^2^ = 0.996; N = 10). Here, j represents the absolute reduction *I*_p_ density, which is obtained by normalizing the steady-state I in (a) to the electrochemically estimated electrode surface area. Conversely, in the range of 10 to 1000 µM, the relationship (c) can be described as j/µA cm^−2^ = 0.0444 ± 0.0016 C/μM + 30.7 ± 0.47 (R^2^ = 0.972; N = 25). Therefore, the sensitivity values for the two linear ranges were determined to be 0.42 and 0.044 mA mM^−1^ cm^−2^. The data acquired from the 2D sensors are illustrated in (d), which consists of two linear segments: (e) ranging from 0.2 to 10 µM and (f) ranging from 10 to 1100 µM. To comprehend the presence of two linear associations, an examination was conducted on the dynamic range of H_2_O_2_ both using an HRP-modified glassy carbon electrode and without HRP. The chronoamperometric findings obtained from 0.1 to 1 µM at the HRP-modified glassy carbon electrode, as illustrated in (g), were analyzed. However, the chronoamperometric responses of 0.1 M PBS at the glassy carbon electrode without HRP, as seen in (h). As seen in (i), the *I* response remained mostly unaffected by the interfering chemicals, in contrast to the notable impact observed with 0.05 mM H_2_O_2_. Therefore, it is expected that this biosensor will serve as an essential tool in facilitating the prognosis and advancement of treatment for oxidative stress disorders resulting from mitochondrial abnormalities.

In related work, Wang et al. presented a novel and simplified methodology for the identification of ROS, which are end products of metabolism that have strong correlations with human diseases. The application of polydopamine (PDA)-covered reduced graphene oxide (rGO@PDA) was proposed by the authors as an alternative to pure graphene in the synthesis of ultrathin 2D graphene-like CeO_2_-TiO_2_ mesoporous nanosheets (MNS-CeO_2_-TiO_2_) (Figure 5C) [101]. The ease of metal precursor loading onto the template is enhanced by the adsorption of metal ions by PDA. Furthermore, the synthesis of AgNPs on a composite material consisting of mesoporous silica (MNS), CeO_2_, and TiO_2_ is accomplished via an in situ photocatalytic reduction process. This leads to the production of a novel composite material known as Ag/MNS-CeO_2_-TiO_2_, which is then employed in the construction of an O_2_-detecting EC sensor. The sensor exhibits successful results, including a sensitivity of 737.1 µA cm^2^/mM and an LOD of 0.0879 µM. The MNS-CeO_2_-TiO_2_ nanomaterial is anticipated to exhibit high efficiency as an enzyme-mimicking catalyst for the dismutation of O_2_^•−^.

After the proposed biosensor was fabricated, the EC performance of the aforementioned materials was investigated by modifying the synthesized materials onto SPCEs to create modified SPCEs. The first step was conducting a CV analysis. Additional evidence about the function of each constituent in Ag/MNS-CeO_2_-TiO_2_ was provided from the amperometric reactions of Ag/MNS-TiO_2_/SPCE, MNS-CeO_2_-TiO_2_/SPCE, and Ag/MNS-CeO_2_-TiO_2_/SPCE toward 0.08 mM O_2_, as seen in Figure 5D. In comparison to Ag/MNS-CeO_2_-TiO_2_, the dispersed AgNPs of the prepared composite resulted in an enhanced I signal, hence improving the sensitivity for the detection of O_2_. Furthermore, in the absence of the superoxide mimetic enzyme component, the Ag/MNS-TiO_2_/SPCE without CeO_2_ exhibited a drop in the efficiency of O_2_ dismutation and a reduction in the reduction I.

Next, the EIS technique was employed to examine the electron transfer efficiency of the produced electrodes. In Figure 5E, it can be shown that the resistance of the MNS-CeO_2_-TiO_2_-450/SPCE was greatly reduced compared to the SPCE. This may be attributed to the further enhancement of the ultrathin 2D porous structure of the MNS-CeO_2_-TiO_2_-450/SPCE. Figure 5F reveals a progressive reduction in the *I* response of the Ag/MNS-CeO_2_-TiO_2_-X/SPCE (X = 450, 600, 800) toward O_2_ as the annealing temperature for the preparation of MNS-CeO_2_-TiO_2_ increased. The authors then introduced an equivalent quantity of O_2_ (0.08 mM) inside the specified potential window ranging from 0.25 to −0.65 V. Consequently, the Ag/MNS-CeO_2_-TiO_2_-450/SPCE composite demonstrated notable sensitivity levels when measured at the potentials of 0.55 and 0.65 V. Under conditions of ideal potential, it was discovered that the reduction I exhibited a simultaneous increase with the constant introduction of O_2_ (Figure 5G).

The evaluation of the electrode performance in practical applications necessitates careful consideration of selectivity, reproducibility, and stability, which are crucial factors in determining the performance of decorated sensors in biosensing. Figure 5H illustrates the experimental design where O_2_ (at 0.08 mM) and common interferents were introduced into 0.1 M PBS solution. Physiologically appropriate concentrations in serum and cell release were considered. The resulting *I* responses were then measured and recorded. The fabricated sensors exhibited minimal reaction to interfering substances, thereby confirming their exceptional selectivity. This EC biosensor is expected to create opportunities for the advancement of biosensing materials and pertinent technologies.

The main aim of developing EC sensors for ROS detection is focused on the medical field, as previously stated. The incorporation of these methodologies into clinical research is expected to facilitate the monitoring of treatment progress. Furthermore, the presented methodologies can potentially be utilized to investigate the antioxidant activity of biologically active compounds or novel pharmaceuticals. In our view, the primary goal of investigations aimed at developing electrochemical sensors for detecting ROS should be the establishment of robust, renewable, and replicable systems.

### 3.3. EC Detection of Glutamate and ATP

Glutamate functions as the primary excitatory neurotransmitter within the central nervous system of vertebrates, exerting an important influence on many physiological and pathological brain activities [102]. Moreover, a decrease in glutamate levels can lead to neuronal damage, such as the development of Alzheimer’s disease [103], and compounds can naturally occur that are particularly high in proteins. Various analytical techniques have been employed thus far to quantify glutamate, including chromatographic, spectrophotometric, capillary electrophoresis, optical, potentiometric, and fluorometric approaches. Nevertheless, the use of these conventional techniques necessitates the acquisition of expensive apparatus, laborious procedures for sample preparation, and the expertise of skilled individuals to operate them. Commonly, electrochemical biosensors are employed for the detection of glutamate [85,86] in order to diagnose a wide variety of diseases.

**Figure 6:**
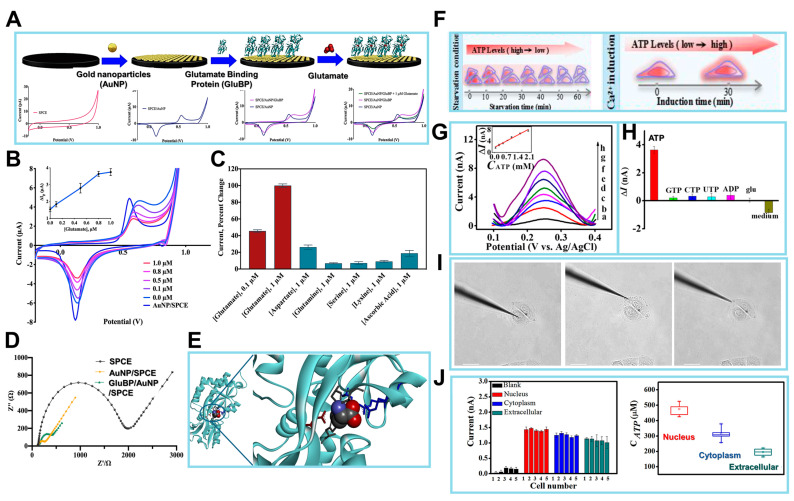
(**A**) Schematics of the surface of the working electrode in the process of the fabricated biosensor. Analytical performance of the GluBP-based biosensor. Cyclic voltammograms of SPCE, AuNP/SPCE, and GluBP/AuNP/SPCE in phosphate buffer (PB), and GluBP/AuNP/SPCE in the presence of 1 μM glutamate in PB. Scanning range is from −0.2 to +1.0 V vs. Ag/AgCl at a rate of 50 mV/s in 50 mM PB, pH 7.4. (**B**) Cyclic voltammograms of AuNP/SPCE in 50 mM PB (pH 7.4) and GluBP/AuNP/SPCE in the presence of different glutamate concentrations (0.0, 0.1, 0.5, 0.8, and 1.0 μM) in the same buffer. Inset: calibration plot for the change in the Au reduction peak current (**I**) vs. glutamate concentration. (**C**) Cross-reactivity of the GluBP-based sensor with other amino acids and ascorbic acid, a common interfering substance. (**D**) EIS characterization of SPCE, AuNP/SPCE and GluBP/AuNP/SPCE in 10 mM [Fe(CN)_6_]^3−/4−^. (**E**) X-ray crystal structure of GluBP35 demonstrating the binding pocket with charged amino acid residues stabilizing glutamate and neutral amino acid residues forming hydrogen bonds with glutamate. (**F**) The nanoaptasensors for the detection of ATP. (**G**) DPV responses of the nanoaptasensor to different concentrations of ATP in 10 mM PBS (pH 7.4), (a–h) 0, 0.05, 0.25, 0.50, 0.75, 1.0, 1.5, and 2.0 mM. Inset: calibration plot of ATP. (**H**) Histograms of the increased peak *I*’s of the nanoaptasensors for 1.0 mM ATP, 5.0 mM ATP analogues, 25 mM glucose, and cell culture medium. (**I**) Representative brightfield images of HeLa cells after nanoaptasensors inserted into different regions (nucleus, cytoplasm, and extracellular space). Scale bar: 20 μm. (**J**) *I* responses to insertion of the nanoaptasensor into different regions of five HeLa cells. Box graph summarizing the average ATP concentrations when nanoaptasensor inserted into different regions of five HeLa cells. (Reprinted with permission from [104]. Copyright 2021, Elsevier; reprinted with permission from [107]. Copyright 2020, American Chemical Society).

To address these obstacles, Zeynaloo et al. devised a mediator-free EC sensor for directly detecting glutamate [104]. This was achieved by immobilizing the periplasmic glutamate binding protein (GluBP) from *Escherichia coli*, which had been genetically engineered, onto SPCEs modified with gold nanoparticles (AuNPs) (Figure 6A). The authors implemented an EC method to investigate the specific binding of GluBP to glutamate and its resulting conformational alteration. Following each stage of biosensor manufacturing, the electrode’s response was evaluated by conducting a potential sweep ranging from −0.2 to 1.0 V, with the scan rate at 50 mV/s. Under these experimental circumstances, it was observed that the electrode surface lacked AuNPs, resulting in the redox-inert behavior of the SPCEs (Figure 6A). Conversely, when the AuNP-modified electrode under otherwise identical conditions was subjected to the process, gold oxide (AuO_x_) was detected on the surface of the electrode at a peak potential of +0.57 V. The reversibility of the AuO_x_ layer development was demonstrated via the reduction of the AuO_x_ during the reverse scan, resulting in the observation of a peak at a potential of +0.13 V.

With the CV response of the AuNP/SPCEs as the reference point, additional tests were performed without GluBP. The relationship between the *I*_p_ intensity of the Au redox reaction and the size and distribution of the AuNPs on the electrode surface, as well as the effective surface area, may be inferred from the cyclic voltammograms. The GluBP protein was genetically modified via the insertion of two cysteine residues at the C-terminal region in order to facilitate the formation of thiol bonds with Au. The active surface area of AuNPs is diminished due to the presence of the non-conductive protein on the electrodes’ surface. Consequently, a drop in the *I*_p_ is detected during the CV analysis of GluBP/AuNP/SPCEs.

The CV response of the GluBP/AuNP/SPCE was then assessed after 30 μL of 1 μM glutamate in phosphate buffer (PB, pH 7.4) was introduced onto the electrode surface, employing identical experimental conditions. This resulted in a further reduction in the *I*_p_ of Au. The CVs were acquired using bare AuNP/SPCE and GluBP/AuNP/SPCE electrodes under conditions both with and without the presence of glutamate, as shown in Figure 6B,C. CVs were obtained throughout the potential range of −0.2 to +1.0 V vs. Ag/AgCl in PB solution (pH 7.4). The scan rate employed was 50 mV/s. The reduction of AuNPs, which is indicated by the *I*_p_, was detected at a potential of +0.13 V on the GluBP/AuNP/SPCE electrode. However, the presence of glutamate during the reverse scan resulted in a decrease in this *I*_p_. A calibration plot was generated to depict the variation in *I*_p_ intensity resulting from the administration of glutamate at different concentrations. The plot revealed a linear range from 0.1 to 0.8 μM, as seen in Figure 6B,C. The LOD of 0.15 was determined. All measurements were conducted using three separate GluBP/AuNP/SPCEs.

While the binding pocket of GluBP has a high affinity for glutamate, it remains plausible that some small molecules may bind to GluBP and induce a physiological reaction. Hence, the constructed biosensor’s reaction was assessed in the presence of several frequently encountered tiny molecules that have the potential to disrupt the accuracy of the results. AA is a frequently encountered interfering component in EC studies conducted on physiological fluids. Lysine, conversely, is an amino acid with a positive charge that possesses the capability to attach to the negatively charged binding pocket of GluBP. Additionally, serine is a small amino acid with polar and neutral characteristics. Glutamine, an amino acid with a polar nature, exhibits structural similarity to glutamate and may have the ability to impede the sensor’s response.

The verification of the protein–gold conjugation was achieved via electrochemical impedance spectroscopy (EIS). Figure 6D displays the Nyquist plots for three different biosensors: the bare SPCE, the AuNP/SPCE, and the GluBP-based biosensor. These plots were obtained under specific conditions. The frequency range used was from 100 mHz to 100 MHz, and the potential applied was +0.18 V (vs. Ag/AgCl). The AC amplitude used was 10 mV. The SPCE exhibited a bigger semicircle compared to the AuNP/SPCE, which can be attributed to the lower conductivity of the bare SPCE in contrast to the AuNP/SPCE. Nevertheless, following the conjugation of GluBP on the electrode’s surface, there was a noticeable augmentation in the diameter of the semicircle in the Nyquist plot. The presence of non-conductive GluBP on the surface of the AuNP/SPCE leads to an increase in electron transfer resistance, which can be attributed to its conjugation. The curve-fitting analysis conducted on the binding data reveals a dissociation constant of 2.4 × 10^−7^ M for the 1:1 binding interaction. An additional chip containing immobilized GluBP was assessed, resulting in an independent determination of a dissociation constant of 2.4 × 10^−7^ M using identical buffer conditions.

Additionally, for the demonstration of the analytical performance for this glutamate biosensor, the X-ray crystal structure of GluBP was analyzed. The selective binding of GluBP to glutamate can be elucidated by examining the charge distribution within the protein’s binding pocket. Three arginine residues are densely arranged with positive charge. The GluBP contains four glutamic acid residues and two aspartic acid residues that are negatively charged. These residues are distributed across the binding cleft of the GluBP, as shown in Figure 6E. The typical charge of the binding pocket is negative charge, which can prevent most of the negatively charged molecules from attaching to GluBP. The charge distribution within the binding pocket is such that the amino group of glutamate is located in the negatively charged zone, while both carboxylate groups are located in the positively charged region. The binding of GluBP to glutamate entails a complex process that occurs in multiple states. When the protein is fixed on the surface of the AuNP/SPCEs, the shift of the protein’s shape from an open to a closed state upon binding to glutamate causes a modification of the electrode’s surface. This modification may be detected via electrochemical measurements.

The novel platform developed in this study for the detection of analytes was achieved by integrating a periplasmic binding protein, GluBP, which exhibits a substantial change in its conformation upon interacting with the analyte. The GluBP-based biosensor, which did not need the presence of enzymes, demonstrated efficacy in quantifying glutamate levels under physiological pH conditions. It exhibited a low LOD and a reasonably rapid reaction time. The sensor exhibited a response to glutamate that was dependent on the dose, demonstrating a linear relationship within the concentration range of 0.1 to 0.8 μM, as demonstrated via CV. This GluBP biosensor system exhibits remarkable promise as a versatile platform for the detection of glutamate in various biological samples. Future research in the advancement of glutamate sensors should focus on utilizing nanostructures composed of readily available materials that can directly transfer electrons with glutamate without the need for enzymes or mediators. These sensors should be customizable to be applicable in vivo and in the food industry.

ATP is the main energy carrier in biological organisms, playing an essential part in cellular metabolism pathways and several biochemical reaction processes [105]; thus, intracellular ATP serves as a reliable biomarker for the presence and level of biological activity in living organisms [106]. To this end, considerable work has been carried out to detect ATP at the subcellular scale in the context of cellular energy metabolism using electrochemical method on various modified electrodes [88,89]. Specifically, to enhance their sensitivity, aptamers were used for ATP detection. Then, to assess their electrochemical sensing, electrocatalytic material (ferrocene) or DNA were used for ATP sensing. In fact, Zheng et al. developed an EC nanoaptasensor for monitoring ATP fluctuations at the subcellular level that embraces the features of easy fabrication, specificity, and outstanding biocompatibility [106]. A Au nanoelectrode was successfully manufactured, possessing a diameter measuring 120 nm. The nanoelectrode surface was decorated with ferrocene (Fc)-labeled anti-ATP aptamer; here, Fc was selected as a biological recognition element, and the anti-ATP aptamer with a strong affinity for ATP was selected as an electroactive reagent. The Fc-ATP aptamer underwent a structural change in the presence of ATP, leading to the closeness of Fc to the nanoelectrode surface. Consequently, there was an observed increase in the EC oxidation I of Fc. ATP fluctuation at the subcellular level was measured during Glc deprivation and Ca^2+^ induction by applying a sensing method based on a silanized nanopipette with a tip diameter of 60 nm and an inner cone angle of 4.0°, as shown in the field emission scanning electron microscopy (FE-SEM) image. Following the deposition of a Au layer, it was observed that the tip diameter exhibited an increase to 120 nm, accompanied by a corresponding rise in the inner cone angle to 4.5°. The presence of a Au layer on the surface silanized nanopipettes was confirmed through elemental mapping analysis using the high-angle annular dark-field SEM imaging mode. Furthermore, the FE-SEM image reveals that the Au nanoelectrode, which has been insulated using epoxy resin, exhibits the formation of a Au nanocone tip. This nanocone tip, measuring 0.12 mm in length, functions as an active electrode.

Subsequently, the analytical performance of the sensor was analyzed by evaluating the detection of various concentrations of ATP through differential pulse voltammetry (DPV) experiments (Figure 6G). The nanoaptasensor exhibited a minor oxidation peak at +0.25 V and a corresponding I of 0.89 nA in the absence of ATP. This observation can be attributed to the oxidation of Fc on the Au nanoelectrode. The presence of a low background I is advantageous in achieving very sensitive detection of targets, a characteristic that may be attributed to the diminutive dimensions of the Au nanoelectrode. The observed trend in Figure 6G (inset) indicates that the *I*_p_ exhibited a progressive rise when the ATP concentration was increased within the range of 0.05–2.0 mM. This concentration range is known to be representative of cATP levels. The equation for linear regression may be expressed as ΔI (nA) = 3.12 cATP (mM) + 1.83, with a correlation coefficient of 0.9864. The LOD for this equation is determined to be 19 μM. An RSD of 3.8% was observed for a single nanoaptasensor when five repeated measurements were conducted. In contrast, an RSD of 6.3% was achieved when five distinct nanoaptasensors were used to measure 0.25 mM ATP.

To assess the specificity of the nanoaptasensors, the nanoaptasensors were reacted with cytidine 5′-triphosphate, guanosine 5′-triphosphate, adenosine 5′-diphosphate, uridine 5′-triphosphate, Glc, and cell culture media, respectively. A detectable I for these reactions is not readily apparent in contrast to the reaction seen at a concentration of 1.0 mM ATP, as depicted in Figure 6H. Another attractive feature of the sensor was the 90.3% retention of the I after refrigerated storage at 4 °C for 7 days.

Inspired by the favorable biocompatibility and analytical performance of the nanoaptasensor, the assessment of ATP content at the subcellular scale was conducted via the precise insertion of the said nanoaptasensors into the nucleus, cytoplasm, and extracellular space of HeLa cells (Figure 6I). The *I* responses obtained from the nanoaptasensors placed into various subcellular sites are depicted in Figure 6J. The nanoaptasensor, when inserted into the nucleus, cytoplasm, and extracellular space of each HeLa cell, exhibited a larger *I*_p_ compared to its introduction into the cell culture medium devoid of cells. The discovery suggests that the DPV approach does not encounter any interference by electroactive compounds in cellular liquids at +0.25 V. The observed EC reaction is attributed to the presence of Fc on the electrode surface and the existence of ATP in cells. The *I*_p_ seen in various cells within the same areas exhibits a minor variation, ranging from 8% to 17% RSD. This discrepancy might be attributed to the inherent heterogeneity present among the cells.

The investigation also encompassed the examination of the regeneration process of the nanoaptasensors following their repeated implantation into cellular entities. After three insertions and two regenerations into various cellular locations, including the extracellular space, cytoplasm, and nucleus, the obtained values for the first enhanced I_p_ were 93%, 89%, and 89%, respectively. The nanoaptasensors exhibiting a CV I_p_ below 6% were chosen for subsequent use in cell-based assays. Nanoaptasensor measurements of ATP content in five individual cells were about 439 ± 37, 306 ± 42, and 198 ± 35 μM in the nucleus, cytoplasm, and extracellular space, respectively. As this study highlights, the integration of nanoelectrodes with aptamers enables fulfilling of the criteria for examining ATP-related biological processes using EC techniques at the subcellular scale. The application of this technique has the potential to expand the identification of various biological compounds at the subcellular scale, and the nanoelectrode ensures a high spatial resolution. Electrochemical detection of ATP using ATP-sensing biosensors is currently helping in gaining a deeper understanding of purinergic signaling and enabling early illness diagnosis, in addition to the well-established instrumental techniques and biochemical analysis, and will continue to do so.

## 4. Applications of EC Biosensors for Monitoring Metabolic Reactions

Cells are intricate biological entities that play a crucial role in carrying out numerous biological functions [108]. Hence, the examination of the fundamental features of these entities, including growth, migration, differentiation, and viability, is of utmost value in seeking novel therapeutic approaches for the diagnosis and treatment of diverse ailments. To achieve precise diagnosis and detection, it is important to ascertain the dynamics of metabolic processes. In this section, we discuss the various applications of EC biosensors for monitoring metabolic reactions (Table 3).

**Figure 7:**
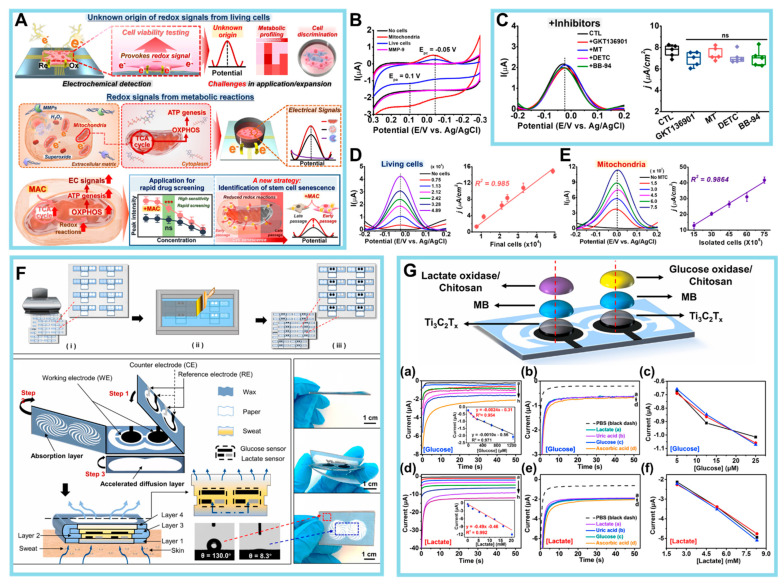
(**A**) Origin of redox signals in live cells and versatile applications. (**B**) CV signals obtained from isolated mitochondria, HeLa cells, and MMP-9. (**C**) DPV signals from HeLa cells treated with MMP inhibitors: 2 μM GKT136901, 2 μM Mito-Tempo (MT), 10 μM sodium diethyldithiocarbamate (DETC), and 10 μM batimastat (BB-94). Box plot of the calculated current (*I*) densities measured in the left panel (*j* indicates the *I* densities). (**D**) DPV signals with varying numbers of HeLa cells ranging from 7466 to 48,866 cells. Linear correlations (R^2^) of the calculated *I* densities from the DPV graph and final number of HeLa cells cultured on the HCGN platform. (**E**) DPV signals (E_p_ = −0.02 V) elicited from isolated mitochondria with various numbers of cultured HeLa cells. Linear correlations (R^2^) of the calculated *I* densities from the DPV graph and the mitochondria isolated from HeLa cells. (**F**) Schematic illustration and photos of the HIS paper. (**G**) The sensor properties of the HIS paper. (**a**) Amperometric response and calibration curve of the glucose (Glc) sensor with Glc concentrations of 0, 0.075, 0.15, 0.25, 0.45, 0.65, 0.95, and 1.25 mM in 60 μL of 0.1 M PBS (pH 6.71), −0.18 V. Inset: the calibration curves of Glc. (**b**) Interference study in the presence of 0.15 mM Glc (plot c), followed by subsequent 50 μM additions of lactate (plot a), uric acid (plot b), and ascorbic acid (plot d). (**c**) Reproducibility of the Glc sensors manufactured in different batches at Glc concentrations of 0.15, 0.25, and 0.45 mM. (**d**) Amperometric response and calibration curve of the lactate sensor with lactate concentrations of 0, 0.3, 2.3, 5.3, 8.3, 12.3, 16.3, and 20.3 mM in 60 μL of 0.1 M PBS (pH 6.71), −0.20 V. Inset: the calibration curves of lactate. (**e**) Interference study in the presence of 2.0 mM lactate (plot a), followed by subsequent 50 μM additions of uric acid (plot b), Glc (plot c), and ascorbic acid (plot d). (**f**) Reproducibility of the lactate sensors manufactured in different batches at lactate concentrations of 2.3, 5.3, and 8.3 mM. (Reprinted with permission from [115]. Copyright 2023, Wiley; reprinted with permission from [116]. Copyright 2021, Elsevier).

The first discovery made by Koo et al. pertained to the identification of intracellular metabolic pathways as the primary sources of EC signals that may be detected extracellularly [115]. This finding was supported by Figure 7A in their study. To ascertain the source of redox signals within live cells, the researchers initially directed their attention toward a biological constituent that exhibits proximity to the electrode surface during EC examination. The focus of the study was matrix metalloproteinases (MMPs), which are enzymes responsible for the degradation of extracellular matrix proteins. MMPs were selected due to their mode of action involving the hydrolysis of peptide bonds, resulting in proteolytic degradation. This mechanism may be detected using potentiometric techniques. In their investigation, MMPs were individually immobilized on the constructed cell chip at different concentrations ranging from 0 to 200 ng/mL. This immobilization was achieved using EDC/NHS coupling chemistry, and further EC detection was conducted. According to the data shown in Figure 7B, no signals were detectable through CV analysis. This finding contrasted with the signals exhibited by live cells, which demonstrated an increase in signal intensity that was dependent on the concentration.

Subsequently, the authors focused on ROS because of the susceptibility of ROS to oxidation under the influence of electrical force, which renders them observable via EC studies. Nevertheless, it should be noted that ROS exhibit a high level of reactivity and exist in such transient states that their free radical forms cannot be identified for a significant duration. The researchers aimed to indirectly ascertain the cell activity by employing three ROS inhibitors: GKT136901, an inhibitor of NADPH oxidase I/IV, to prevent excessive ROS production; Mito-Tempo, a combination of antioxidants designed to specifically target mitochondria; and sodium diethyldithiocarbamate, an inhibitor of superoxide dismutase. Based on the findings depicted in Figure 7C, it can be concluded that there were no discernible alterations in the signal, suggesting that the formation of ROS and their subsequent oxidation did not play a major role in modulating intracellular redox signals. Finally, mitochondria, which are an internal organelle characterized by their distinct membrane and assortment of redox proteins associated with the ETC, were also analyzed. The isolated mitochondria exhibited a notable linear correlation with the electrical I density (R^2^ = 0.9864), demonstrating consistency with the signals seen in viable cells (R^2^ = 0.985) (Figure 7D,E).

Recently, there has been a notable increase in the efforts of several prominent pharmaceutical firms to explore the development of novel therapeutic compounds with the ability to specifically interfere with mitochondrial processes and impair the functioning of cancer cells. In the context of stem cell research, there exists an urgent demand for innovative methodologies that can effectively measure the multipotency, differentiation, and function of stem cells without the need for labeling or causing cell damage. Considering the strong association between cellular activities and metabolic reactions, the revelation of the source of cellular redox signals and the suggested EC approach will have important implications beyond the development of innovative anticancer medications.

For the purposes of developing a wearable EC biosensor to detect Glc for real-time monitoring, Li et al. [116] devised a sophisticated sensing paper known as highly integrated sensing paper (HIS paper). This innovative paper incorporates 2D Ti_3_C_2_T_x_ as the active material and foldable all-paper substrates as a sweat analysis patch. Notably, the HIS paper integrates a signal processing system for the simultaneous detection of lactate and Glc, thereby enabling real-time analysis of sweat. The various functional components of the HIS paper were printed onto a paper substrate and subsequently folded to create a 3D structure. Through the incremental enlargement of the hydrophilic area, the capillary-driven pressure forced the perspiration to permeate into the paper substrate vertically. The arrangement of the working electrode, counter/reference electrodes, and their respective 3D structures enabled the attachment of enzymes and EC materials onto the working electrode. This arrangement also enhanced the ability to detect perspiration. The single-layer structure of Ti_3_C_2_T_x_ served as a very sensitive interface, enabling the easy immobilization of biomolecules. Furthermore, methylene blue-modification on Ti_3_C_2_T_x_ enhanced the migration of charges, leading to improved EC performance in the sweat analysis. The all-paper sweat sensor possessed several notable advantages, including affordability, compact size, and user-friendly characteristics. The distinctive 3D structural design facilitated the absorption of perspiration from the surface of the skin, preventing the formation of fluids and thus mitigating the discomfort caused by sweat at the contact between humans and machines. Furthermore, the optimization of sweat absorption enhances the mitigation of sweat accumulation on the electrode surface, hence facilitating the reception of signals originating from immediate perspiration rather than accumulated perspiration. Consequently, this enhancement contributed to the amelioration of the precision in promptly detecting the composition of sweat.

HIS paper demonstrates adequate sensitivity in the detection of various biomarkers, specifically lactate and Glc. Figure 7G(a) illustrates the amperometric reactions of HIS paper in response to various Glc concentrations. The data were gathered for 30 s for each release in the HIS area. In the inset of Figure 7G(a), two calibration curves are depicted. These curves demonstrate that the chronoamperometric I’s of Glc exhibit linear correlations with the Glc concentration within the range of 0.08–1.25 mM. The sensitivities were determined to be 2.4 nA/μM at the low concentration and 1.0 nA/μM at the high concentration. The LOD was determined to be 17.05 μM. Hence, the sensitivity and linear range of the Glc sensor make it suitable for the detection of Glc in actual sweat samples. The amperometric response of the lactate sensor to various lactate concentrations within the physiological range of 0.3–20.3 mM is illustrated in Figure 7G(d). The linear response of the sensor to lactate concentrations within the specified range is supported by the data presented in Figure 7G(d). The sensitivity of the sensor in this range was determined to be 0.49 μA/mM, and the LOD was calculated to be 3.73 μM. The DPV test was conducted to examine the linear connection between the Glc and lactate sensors in terms of respective marker concentrations.

While the findings obtained using DPV exhibit heightened sensitivity, it is important to acknowledge the presence of significant errors in both the baseline correction and fitting procedures applied to the DPV data. Hence, the outcomes obtained from the chronoamperometry fits are used for the determination of sensor sensitivity and LOD. The degree of selectivity exhibited by the sweat sensor has considerable importance due to the influence of electrolytes and metabolites present in sweat, which directly impact the precision and reliability of the sensor’s measurements. The impact of non-target electrolytes and metabolites on the response of each sensor is minimal. Figure 7G(b,e) demonstrates the notable selectivity of the proposed sensor toward biomarkers in the presence of AA, UA, and lactate or Glc. There is a lack of discernible interference signals. This implies that the sensor exhibits favorable selectivity, hence facilitating the development of a multiplexed sensing system.

Furthermore, it is important to consider the aspects of repeatability and long-term stability when considering the commercial applications of wearable sensors. The results presented in Figure 7G(c,f) indicate that the sensors produced in distinct batches had comparable responses, thus highlighting the favorable repeatability of these sensors. The sensor exhibits minimal variation in its EC performance over 14 days, indicating a high level of periodic stability. Sweat commonly has a pH range of slightly acidic to neutral, generally ranging from pH 4.5 to 7.0. The temperature of the human skin exhibits variation, typically falling between 33.5 and 36.9 °C. Results obtained from experiments conducted to investigate the impact of pH levels and temperature on the performance of the paper-based sensor for the detection of heavy metal ions demonstrated the effect of pH and temperature variations on the EC performance of a Glc sensor operating with a Glc concentration of 0.5 mM, as well as a lactate sensor operating with a lactate concentration of 5 mM. There was a marginal change of 2.43% and 1.35% in response to variations in pH and temperature, respectively. The findings indicate that the sensing performance is hardly affected by variations in pH and temperature. In practical situations, the mitigation of this impact can be achieved by calibrating the data under varying pH and temperature conditions.

In the future, from our perspective, commercial devices may necessitate the implementation of methods to easily substitute sensing components (such as disposable and inexpensive chemical sensors) with reusable electronic components. Nevertheless, the incorporation of novel attributes such as self-healing, biocompatibility, and biodegradability ensures advancements in convenience and user comfort, hence necessitating the development of next-generation wearable sensors.

## 5. Conclusions and Future Perspectives

This paper provides a comprehensive overview of the successful EC biosensors developed for the detection of cell energy metabolism pathways, such as glycolysis, as well as mitochondrial metabolites. Additionally, it explores the diverse applications of these biosensors in wearable sensors and cell chips for the detection of various metabolites. The EC approach described herein is a highly effective and accurate analytical technique that offers a non-invasive assessment of a wide array of materials under investigation. Various peptides, aptamers, and nanomaterials have been applied to improve the sensitivity of sensors, owing to their versatility and functional attributes. The observable EC signal observed during measurement is the outcome of the interactions between specific probes or composites and target metabolites. Therefore, this EC method for the investigation of cell energy metabolism offers precise, rapid, and non-labeled sensing capabilities, which may be employed in diagnostic procedures for metabolic disorders and advancements in the pharmaceutical field. The incorporation of nanomaterials into EC biosensors has enabled the monitoring of dynamic cell energy metabolism. This includes the accurate detection of glycolytic and mitochondrial metabolites such as Glc, lactate, NADH, ROS, glutamate, and ATP. Furthermore, these biosensors allow for real-time in situ observation of the redox activities associated with these metabolites.

Biosensing technologies that involve electrical and EC systems have shown immense promise as cost-effective, expedient methods for medical diagnostics and POC applications in the future [117,118]. Nevertheless, various challenges continue to exist despite the extensive research carried out on the subject. For instance, it is necessary to improve both longevity and sensitivity. Additionally, it is crucial to acknowledge that the commercialization process necessitates the use of metabolite sensors other than glucose sensors. In addition, to overcome current challenges (e.g., longevity and sensitivity), EC biosensors should be integrated and utilize various functional materials and polymers in the electrodes and system. The rapid advancement of EC biosensor technology highlights their future applications in the field of biological research, particularly in the domain of metabolic analysis. The identification of diverse metabolites is attainable, ranging from glycolytic and mitochondrial metabolites to those at the cellular level, which correlate with metabolic activity. Furthermore, these approaches offer notable gains in sensitivity and running time, hence presenting potential benefits for the future. Hence, the integration of EC biosensors for the detection of cell energy metabolism has the potential for incorporation into developed large-scale systems aimed at early disease diagnosis and facilitating metabolic investigations in the broader biological domain.

## Figures and Tables

**Table 1:** Electrochemical biosensors for glycolytic metabolite detection.

Detection Method	Metabolite	Electrodes	Linear Range	Sensitivity (µA/mMcm^2^)	Stability	LOD	Ref.
Amperometry	Glucose	GOx/AuLr-TiND	0.04–40 mM	10.63 ± 1.28	~5 days (5 mM of glucose)	1.75 ± 0.30 μM	[59]
Amperometry	Glucose	CuSn/CNF/GCE	0.1–9000 μM	291.4	~30 days (0.5 M glc and 0.15 M NaOH)	0.08 μM	[60]
CV	Glucose	FTO-CNTs/PEI/ GOx	0.07–0.7 mM	63.38	~14 days	70 µM	[61]
Amperometry	Glucose	CuO/ZnO-DSDSHNM	500 nM–100 mM	1536.80	~15 days (GCEs in the air)	357.5 nM	[62]
Amperometry	Lactate	NiCo-LDH/SPCE	5–25 mM	30.59 ± 0.34	~28 days	0.53 mM	[63]
Amperometry	Lactate	PdCu/LIG	0.1–30 mM	−51.91	-	0.28 μM	[64]
CV	Lactate	Ti_3_C_2_@Eu-SnO_2_	1 nM–10 mM	4.815	~13 days	0.338 nM	[65]
Amperometry	Glucose /Lactate	LIG/H-NPC/PB/ GOx	0.3 μM–1.5 mM (Glucose) 0–56 mM (Lactose)	82.7	~7 days	0.025 μM (Glucose) 4 μM (Lactose)	[66]

**Table 2:** Electrochemical biosensors for mitochondrial metabolite detection.

Detection Method	Metabolite	Electrodes	Linear Range	Sensitivity (µA/mMcm^2^)	Stability	LOD	Ref.
Amperometry	NADH	NPG/Os(bpy) 2PVI/DIA	5–100 µM	89.6	~7 days	0.8 µM	[84]
Amperometry	Glutamate	GLDH/Chit-AA-CDs/SPCE	11–125 µM	2.7	~14 days	3.3 µM	[85]
Amperometry	Glutamate	Co_3_O_4_ nanocubes/SPE	10–600 µM	20.12	~30 days	10 µM	[86]
Amperometry	H_2_O_2_	AuNFs/ Fe_3_O_4_@ZIF-8-MoS_2_	5 μM–120 mM	-	~7 days	0.9 μM	[87]
Amperometry	ATP	Aptamer-CFNEs	0.05–2.0 mM	4.341	~7 days	3.4 μM	[88]
SWV	ATP	Aptamer/ 2D DNA structure	1 pM–00 μM	-	~16 days	0.3 pM	[89]
DPV	Cytochrome *c*	Apt/GOAsp/CNF/ GCE	10 nM–100 µM	-	~21 days	0.74 nM	[90]
Amperometry	Cytochrome *c*	Implanted Au wire	0.2–0.8 μM	42.4	~7 days	2.4 nM	[91]

**Table 3:** Applications of electrochemical biosensors for sensing metabolic reactions.

Detection Method	Targets	Wearability	Electrodes	Linear Range	LOD	Ref.
Amperometry	Glucose/Lactate	Forehead patch	PEN/SFNSs/Pt-G	0–4 mM	2 μM	[109]
LSV	Glucose	Hydrogel patch	Pt/MXene hydrogel	0–8 mM	29.15 μM	[110]
Amperometry	Glucose	Arm patch	GOx/Pt-HEC/LSG	5–3000 μM	0.23 μM	[111]
Amperometry	Glucose	Chip-implanted	PPG/GOx/PU	1–30 mM	-	[112]
DPV	Mitochondrial activity	-	AuNP/ITO	4825–31,125 cells	4433 cells	[113]
DPV	Kidney organoid	-	AuNP/ITO	21,000–157,000 cells	21,363 cells	[114]

## Abbreviations

2D: two-dimensional; 3D, three-dimensional; AA, ascorbic acid; AgNP, silver nanoparticle; AgPNP, silver nanoprism; AgRNP, silver nanorod; AgSNP, silver nanosphere; ATP, adenosine 5′-triphosphate; Au, gold; AuNP, gold nanoparticle; AuOx, gold oxide; CNT, carbon nanotube; CV, cyclic voltammetry; DA, dopamine; DPV, differential pulse voltammetry; EC, electrochemical; EDC, 1-ethyl-3-(dimethylaminopropyl)carbodiimidehydrochloride; EIS, electrochemical impedance spectroscopy; ETC, electron transport chain; FAD, flavin adenine dinucleotide; Fc, ferrocene; FE-SEM, field emission scanning electron microscopy; GA, gallic acid; GEDP, glucose electrochemical detection platform; Glc, glucose; GluBP, glutamate binding protein; GO, graphene oxide; GOx, glucose oxidase; HepG2, hepatocellular carcinoma; HIS paper, highly integrated sensing paper; HRP, horseradish peroxidase; *I*, current; *I*_p_, peak current; ISF, interstitial fluid; LOD, limit of detection; LOx, lactate oxidase; MA, microneedle array; MMP, matrix metalloproteinase; MNS, mesoporous silica; NADH, nicotinamide adenine dinucleotide; NHS, *N*-hydroxysuccinimide; NADPH, nicotinamide adenine dinucleotide phosphate; NPQD, 4′-mercapto-*N*-phenylquinone diamine; O_2_^•−^, superoxide anion; ^•^OH, hydroxyl radical; OXPHOS, oxidative phosphorylation; PAAm, polyacrylamide; PANI, polyaniline; PBNP, Prussian blue nanoparticle; PBS, phosphate-buffered saline; PCP, pro-collagen C proteinase; PDA, polydopamine; PEDOT, poly(3,4-ethylenedioxythiophene); PG, pelargonin; POC, point-of-care; PTZ, phenothiazine; R_ct_, resistance to charge transfer; rGO, reduced graphene oxide; RI, reverse iontophoresis; ROS, reactive oxygen species; RSD, relative standard deviation; SA, sodium alginate; SEM, scanning electron microscopy; SPCE, screen-printed carbon electrode; SPE, screen-printed electrode; SPR, surface plasmon resonance; TMSPMA, 3-(trimethoxysilyl)propyl methacrylate; UA, uric acid; ZIF, zeolite imidazole framework.
